# Deciphering Double-Walled Corrugated Board Geometry Using Image Analysis and Genetic Algorithms

**DOI:** 10.3390/s24061772

**Published:** 2024-03-09

**Authors:** Maciej Rogalka, Jakub Krzysztof Grabski, Tomasz Garbowski

**Affiliations:** 1 Institute of Applied Mechanics, Poznan University of Technology, Jana Pawla II 24, 60-965 Poznan, Poland; maciej.rogalka@o2.pl (M.R.); jakub.grabski@put.poznan.pl (J.K.G.); 2 Department of Biosystems Engineering, Poznan University of Life Sciences, Wojska Polskiego 50, 60-627 Poznan, Poland

**Keywords:** corrugated board, double-walled, flute parameters, cross-section images, genetic algorithm

## Abstract

Corrugated board, widely used in the packing industry, is a recyclable and durable material. Its strength and cushioning, influenced by geometry, environmental conditions like humidity and temperature, and paper quality, make it versatile. Double-walled (or five-ply) corrugated board, comprising two flutes and three liners, enhances these properties. This study introduces a novel approach to analyze five-layered corrugated board, extending a previously published algorithm for single-walled boards. Our method focuses on measuring the layer and overall board thickness, flute height, and center lines of each layer. Through the integration of image processing and genetic algorithms, the research successfully developed an algorithm for precise geometric feature identification of double-walled boards. Images were recorded using a special device with a sophisticated camera and image sensor for detailed corrugated board cross-sections. Demonstrating high accuracy, the method only faced limitations with very deformed or damaged samples. This research contributes significantly to quality control in the packaging industry and paves the way for further automated material analysis using advanced machine learning and image sensors. It emphasizes the importance of sample quality and suggests areas for algorithm refinement in order to enhance robustness and accuracy.

## 1. Introduction

Corrugated board, as a material commonly used in the packaging industry [1,2], has many advantages in comparison to the other packing materials. One can notice its strength, lightweight, ease of customization, recyclability, and relatively low costs. It can effectively protect goods during their shipping, storage, and handling. 

In single-walled corrugated boards, the structure consists of one flute and two liners. The latter are often manufactured from kraft paper, a kind of paper that comes from wood pulp. It is known for its durability, strength, and resistance to puncturing or tearing. These properties make it an ideal material for packaging applications, in particular for outer layers like liners. The strength, cushioning, height, or smooth surface of the corrugated board are related to the geometry of the internal layer, that is the flute. The formation of the fluted sheet in the corrugated board involves the paper being fed through a sequence of fluting rollers, resulting in the distinctive ridges and valleys. The higher flutes provide enhanced strength and cushioning, while the smaller flutes are more suitable for printing purposes due to the smoother surface of the resulting corrugated board. The most common flute types are as follows:

- A-flute: its approximate height is 5 mm. The A-flute is commonly used for heavy goods packaging, i.e., furniture, due to its strength and cushioning properties.
- B-flute: its approximate height is 3 mm. The B-flute has quite universal properties. It is very often used for retail packing or shipping boxes.
- C-flute: its approximate height is 4 mm. It is the most commonly used type of flute and has similar applications to the B-flute.
- E-flute: its approximate height is 1.6 mm. It offers a smooth surface, which is appropriate for printing purposes. This type of flute is commonly used for retail packaging and small boxes.
- F-flute: its approximate height is 0.8 mm. It can be applied, similarly to the E-flute, for small boxes and retail packing, providing good printing properties due to smooth surface of the corrugated board.

**Figure 1:**
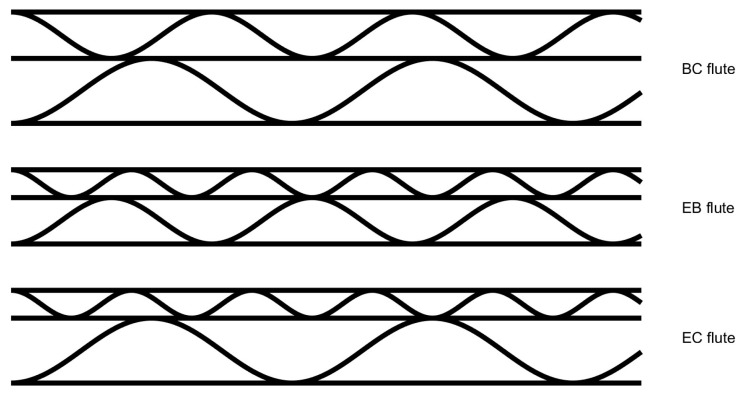
Examples of typical flutes combinations in the double-walled corrugated.

The choice of flute depends on the final specific application. However, it is possible to improve the corrugated board properties by applying the double-walled structure, e.g., to combine cushioning and printing quality or to increase their strength properties. Every kind of flute has particular advantages and is appropriate for certain packing purposes. Manufacturers have the ability to incorporate several flutes in order to produce customized corrugated boards that satisfy particular criteria for strength, cushioning, and printing properties. The double walls available on the market are often composed of BC (5–7 mm), EB (3.5–5 mm), or EC (4–5.5 mm) flutes. Figure 1 presents these examples of flute combinations in the double-walled corrugated board.

The corrugated board is susceptible to warping during both the manufacturing and subsequent stages, such as storage, transit, and usage, which may lead to deformation. The origins of these phenomena are attributed to fluctuations in temperature and humidity, as well as mechanical stresses. There are two sorts of defects in the corrugated board, these include global imperfections and local imperfections. Beck and Ficherauer developed and explained a model that accounts for the organized and extensive bending of the cardboard [3]. The writers of this paper mainly focused on local issues. In 1995, Nordstrand conducted a study to investigate how the magnitude of certain imperfections affects the compressive strength of boxes produced from the corrugated board [4]. In 2004, the author studied local flaws through the examination of the nonlinear buckling of Rhodes and Harvey orthotropic plates [5]. Lu et al. [6] analyzed the mechanical characteristics of corrugated cardboards, explicitly focusing on the effects of imperfections during compression. Garbowski and Knitter-Piątkowska [7] conducted a detailed analysis of the bending properties of double-walled corrugated cardboard. Mrówczyński et al. [8] suggested a technique to analyze single-walled corrugated cardboard through the inclusion of original flaws. Cillie and Coetzee conducted a study on corrugated cardboards that had both global and local defects, subjecting them to in-plane compression [9]. In a recent study, Mrówczyński and Garbowski introduced a straightforward approach to compute the effective stiffness of the corrugated board with geometric imperfections. This technique utilizes the finite element method and the representative volumetric element [10].

Image processing is rarely employed in the study of corrugated boards. Nevertheless, the most prevalent instance is the development of an automated garbage sorting system. Liu et al. created a novel trash classification model using transfer learning and model fusion [11]. Rahman et al. devised a system for categorizing recyclable waste paper based on template matching [12]. A further use of the image processing approach involves calculating the number of layers in the corrugated board. Cebeci used conventional image processing techniques to automate the numbering of the corrugated board [13]. In a similar manner, Suppitaksakul and Rattakorn used a machine vision system and image processing methods to accurately quantify the number of corrugated boards [14]. Subsequently, Suppitaksakul and Suwannakit proposed an algorithm for merging corrugated board pictures [15].

The classification of various materials and cross-section geometrical feature evaluations based on images can be found in the literature. Caputo et al. used the support vector machine algorithm to categorize items via analyzing their photos under different lighting and positioning scenarios [16]. Iqbal Hussain et al. used a convolutional neural network, namely the ResNet-50 architecture, to identify and categorize woven materials [17]. Wyder and Lipson investigated the use of convolutional neural networks to identify the static and dynamic characteristics of cantilever beams using their unprocessed cross-section pictures [18]. Li et al. used a range of deep learning methods to examine the geometric characteristics of a self-piercing riveting cross-section [19]. The authors demonstrated that the SOLOv2 and U-Net topologies provided the most optimal outcomes. Ma et al. examined the geometric characteristics of the crushed cross-sections of thin-walled tubes made of carbon fiber-reinforced polymer [20].

The genetic algorithm is an optimization method that takes inspiration from the natural processes of selection and genetics [21]. These algorithms use the concepts of evolution, including selection, crossover, and mutation. The fundamental concept behind genetic algorithms is to generate a group of individuals that reflect potential solutions to a considering issue. Each individual is represented by a collection of characteristics, referred to as chromosomes or genomes, that may be seen as the genetic material. These chromosomes undergo operations, such as selection, crossover, and mutation, which mimic the genetic processes of reproduction and variation. John Henry Holland [22] is renowned as the founding figure in the field of genetic algorithms, which have shown remarkable effectiveness across various domains including optimization, scheduling, and artificial intelligence. These algorithms are particularly adept at navigating complex, multidimensional search spaces where conventional optimization methods might struggle. In the field of corrugated board production, genetic algorithms have found unique applications. Shoukat combined these algorithms with mixed integer linear programming to optimize cost and greenhouse gas emissions in papermaking [23], while Hidetaka and Masakazu utilized them for scheduling in corrugated board production [24]. In the literature, one can also find some papers related to the use of genetic algorithms in image processing. A review of such applications for image enhancement and segmentation was performed by Paulinas and Ušinskas [25]. Ayala-Ramirez and coauthors employed the genetic algorithms for finding circles in images [26]. Jie et al. applied the genetic algorithm to find elliptic shapes [27]. To the best of our knowledge, only one paper deals with the application of these algorithms for finding flute shapes in images of corrugated boards [28]. However, it was limited to three-layered corrugated boards. This study introduces a significant extension to five-layered corrugated boards. This paper introduces a novel approach to ascertain the geometric features of corrugated boards using a specialized acquisition device and an algorithm that combines image processing with genetic algorithms, focusing on flute geometry. This methodology could lay the groundwork for automatically modeling corrugated board geometry from cross-sectional images. This research stands as an important contribution to the field, offering practical and innovative solutions for the packaging industry. By harnessing the power of genetic algorithms for geometric analysis, it opens new avenues for efficient and accurate corrugated board production, potentially revolutionizing current practices and sustainability in the corrugated packaging sector.

The concepts presented in this paper were initially introduced in our previous article [28]. However, this paper significantly extends the scope and applicability of the earlier research. While the prior study focused on the analysis of three-layered corrugated board structures, the current paper proposes a refined and enhanced algorithm capable of analyzing more complex, multilayered corrugated board structures. Specifically, this new research addresses the challenges associated with five-layered corrugated boards, which hold substantial relevance in the packaging industry. The enhanced algorithm demonstrates improved adaptability and accuracy in dealing with the intricacies of these more complex structures, offering substantial advancements over our previous work.

The paper is organized as follows. Section 2 contains descriptions of the equipment used to acquire the images and the algorithm proposed for the analysis of five-layered corrugated boards. The obtained results are presented in Section 3, and discussed in Section 4. Finally, the conclusions are formulated in Section 5. 

## 2. Materials and Methods

### 2.1. Corrugated Board Cross-Section Image Acquisition

**Figure 2:**
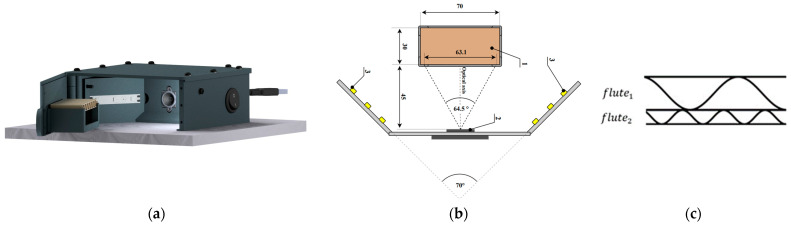
Device for corrugated board image acquisition: (**a**) a 3D model of the device; (**b**) the mutual position of the device components: 1—corrugated board sample; 2—camera; 3—LED strip [Rogalka2023]; (**c**) fluting indexing.

The images of the corrugated board cross-section have been acquired using a device engineered specifically for this purpose. Its precise description can be found in [28]. Images depicting sample cross-sections were taken under uniform conditions, i.e., with controlled LED-sourced illumination and a camera axis perpendicular to the plane of the sample face. Figure 2a presents a 3D model of the device, whereas Figure 2b shows the mutual position of the camera and analyzed corrugated board sample. In the case of the double-walled cardboard, placing the sample in the device holder is necessary to ensure that the higher flute is above the finer one. In the following study, as presented in Figure 2c, the flute located above is referred to as ${flute}_{1}$, and the one below is ${flute}_{2}$.

The device utilizes the ArduCam B0197 camera with a Sony IMX179 (1/3.2″) (Tokyo, Japan) image sensor with a resolution of 8 MPx. The acquired images were saved in a JPEG format at a maximum resolution of 3264 × 2448 pixels.

### 2.2. Algorithm for Corrugated Board Geometrical Feature Identification 

**Figure 3:**
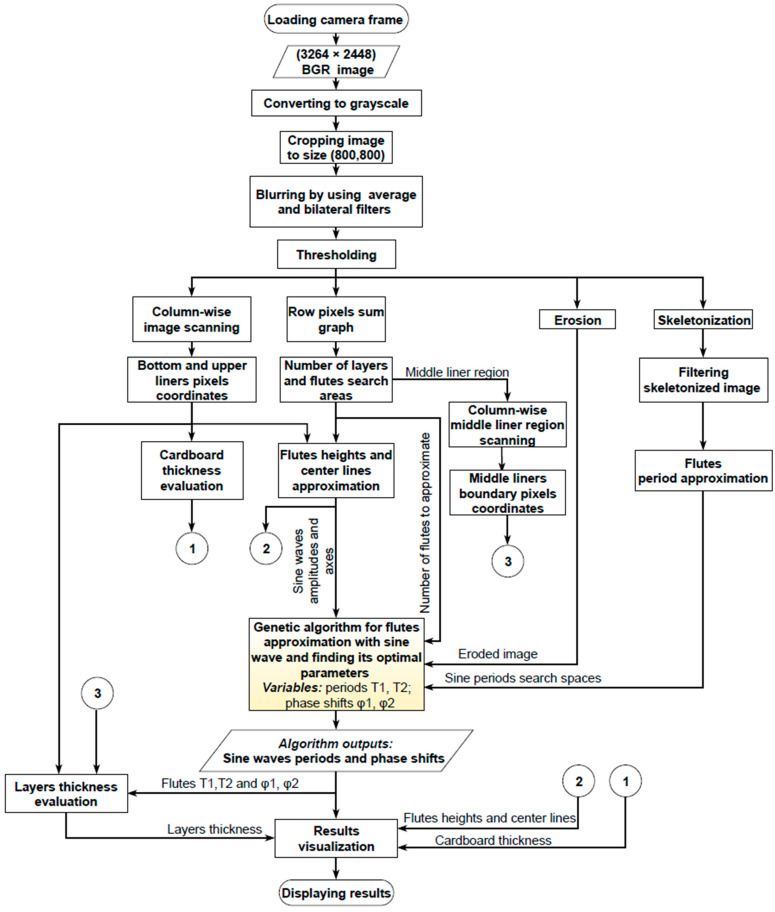
Flow diagram of the proposed method.

Figure 3 presents the flow diagram of the proposed algorithm. The RGB image obtained from the device is first subjected to different preprocessing operations. Various versions of the input image are then utilized in order to identify several geometrical features of a five-layer corrugated board sample, such as its height, the flute heights, periods and phase shifts, and the liner and flute thickness. The algorithm has been implemented in Python 3.9.13 using the OpenCVcontrib-python 4.7.0.68 and geneticalgorithm libraries. 

#### 2.2.1. Image Preprocessing

**Figure 4:**
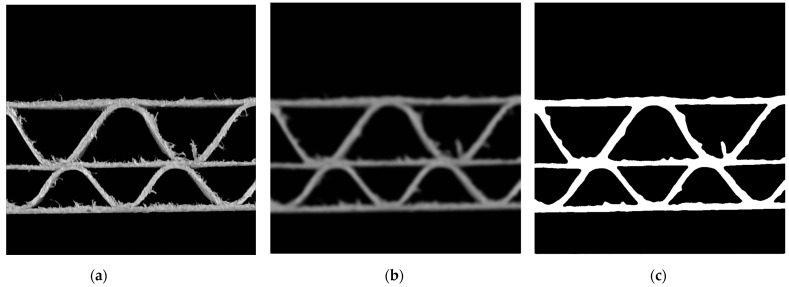
Images obtained as a result of the preprocessing stage: (**a**) a grayscale image cropped to 800 × 800 pixels; (**b**) blurred image; (**c**) binary image.

The input of the system was a single frame from the camera. It was an RGB image with dimensions of 3264 × 2448 pixels. The first preprocessing operation is a grayscale conversion into the range <0, 255>. Next, an 800 × 800 pixels subset of a grayscale image is cut out of the central acquisition area. Figure 4a presents the final image acquired as a result of the described actions. In order to remove small noise from the image (caused by the presence of cellulose fibers), the following two blurring methods were applied: averaging with a normalized box filter and with a kernel size of 3 × 3 and bilateral filter. Figure 4b presents the result of these operations. Finally, the blurred image was converted into a binary image (Figure 4c) by applying a lower threshold binarization with a threshold value equal to 75. All the parameters in the preprocessing stage were chosen empirically.

#### 2.2.2. Corrugated Cardboard Thickness Estimation

**Figure 5:**
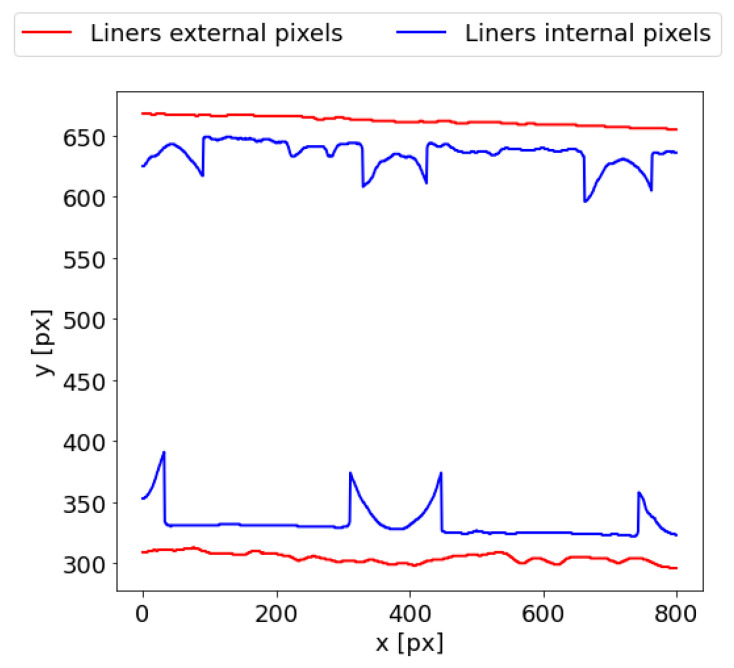
Internal and external pixels coordinates of the upper and lower liners.

The thickness of the five-layer corrugated cardboard can be estimated with the same method used for the three-layer samples which were presented in [28]. The boundary points of the outer liners can be identified by applying column-wise scanning to the binary image (Figure 4c). Pixels in each column of the image are analyzed. The *y* coordinate of the first white pixels in each column is saved to the *ULEP* matrix for scanning from the top of the image towards the bottom. Next, scanning is continued until the first black pixel is recognized. Its *y* coordinate is written in the *ULIP* matrix. As a result, the external points of the upper liner are written in the *ULEP* matrix; the *ULIP* matrix contains upper liner internal points. Analogically, in order to determine lower liner boundary points, the direction of column-wise scanning is reversed, starting from the bottom of the image towards the top. In this way, the new matrices *LLEP* and *LLIP*, which, respectively, store the external and internal pixels of the lower liner, are created. Figure 5 presents the results of this operation. 

In order to determine the corrugated board sample height d, the average distance between the external points of the upper and lower liner are calculated. It can be expressed as

(1) $$d=\frac{1}{NC}\sum_{x=0}^{NC-1}\left|ULEP\left(x\right)-LLEP\left(x\right)\right|,$$

where x denotes the column index, and NC=800 is the total number of columns. 

For the purpose of further geometrical feature identification, the external boundaries of both upper and lower liners are approximated using linear functions and coordinates from the *ULEP* and *LLEP* matrixes. The resulting linear equations can be expressed as

(2) $$y_{U}=a_{U}x+b_{U},$$

(3) $$y_{L}=a_{L}x+b_{L},$$

where $a_{U}$ and $b_{U}$ denote the parameters of the upper liner approximation, while $a_{L}$ and $b_{L}$ are the parameters of the lower liner approximation.

#### 2.2.3. Flutes Center Lines and Heights Estimations

**Figure 6:**
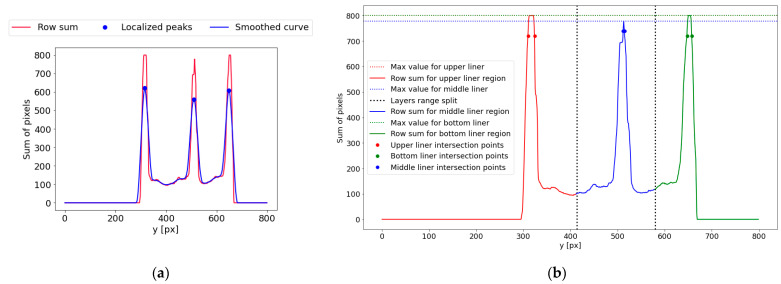
The row sum curve and localizations of the vertical positions of the liners: (**a**) the row sum curve (red line) and the smoothed curve (blue line); (**b**) the upper (red), middle (blue), and lower (green) liner ranges.

At this stage, the corrugated cardboard flutes’ center lines and heights are estimated. The binary image row sum curve is plotted to find the localization of liner and fluting regions. Analyzing the number of white pixels in each row of the image, as presented in [28], allows us to determine the approximate location of the bottom and upper liner in the image. The sample is placed horizontally, and the curve local maximums are related to the presence of flat layers. Therefore, the occurrence of an additional liner in the middle of the sample should create one additional extremum visible on the curve. In the case of the five-layered corrugated board sample, which consists of three liners and two flutes, three local maximums should always be detected at this stage. In order to smooth the row sum curve and highlight the maximum resulting from the liners, the same version of the Savitzky–Golay filter with 30 interpolation points and a first-degree polynomial was applied. Furthermore, the distance between the maximums has to be larger or equal to 20, and the minimal value of the local maximum was equal to 0.4 of the global maximum value. Figure 6a depicts the original row sum and smoothed curves with three local maximums detected. It is also worth noting that the peak values can differ significantly for both the ideal and the creased samples. Their values mainly depend on the overall arrangement of the layers and their thickness.

At this point, the row sum curve can be further analyzed. Based on local maximums, the curve is divided into three ranges. Each range corresponds to the area of one liner. Range borders (black bold dashed line) are determined as middle points between two adjacent local maximums, marked as blue bold dots in Figure 6a. In Figure 6b, the bottom, middle, and upper liner ranges are marked in green, blue, and red, respectively. In each of these intervals, the subsequent actions are carried out:

1. The local maximum $S_{max}$ is identified.
2. The vertical line with an ordinate equal to the value of 0.9$S_{max}$ (for bottom and upper liner regions, or 0.9$5S_{max}$ for middle liner region) is now plotted. Two intersection points of the curve and plotted line are determined and marked by bold dots, as shown in Figure 6b.
3. The distance between the intersection points within each range is calculated and denoted as $b_{US}$, $b_{mS\;}$, and $b_{BS}$ for the upper, middle, and bottom liners, respectively.

**Figure 7:**
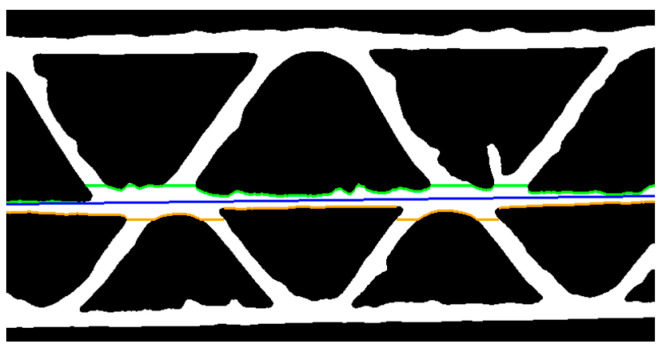
Binary image with the upper (green) and bottom (orange) pixels of the middle liner and its linear approximation (blue).

For the five-layer corrugated board samples, the center line and height estimations are calculated separately for each fluting. First, the middle liner’s approximate location in the image must be determined. Another column-wise scanning of the binary image (Figure 4c) is carried out. The scanning is limited to the rows with coordinates $y\in <y_{int1}-20,y_{int2}+20>$, where $y_{int1\;}$ and $y_{int2}$ are consecutive intersection points of the middle liner visible in Figure 6b. The coordinates of the first white pixels in each column are written into the *MLUP* matrix for scanning from the top to the bottom of the image and for the reversed direction into the *MLBP*. A linear approximation of a line passing through the center of the area bounded by the *MLUP* and *MLBP* pixels is performed. It can be expressed as

(4) $$y_{m}=a_{m}x+b_{m},$$

where $a_{m}$ and $b_{m}$ denote the parameters of the middle liner approximation. Figure 7 shows the results of the above operations.

Both fluting searches can now be limited based on the boundaries of the liners expressed in Equations (2)–(4). The boundary lines limiting the ${fluting}_{1}$ searching can be written in the following forms:

(5) $$y_{1UBL}=a_{U}x+b_{U}+b_{US},$$

(6) $$y_{1LBL}=a_{m}x+b_{m}-b_{mS},$$

whereas for limiting the ${fluting}_{2}$,

(7) $$y_{2UBL}=a_{m}x+b_{m}+b_{mS},$$

(8) $$y_{1LBL}=a_{L}x+b_{L}-b_{LS}.$$

**Figure 8:**
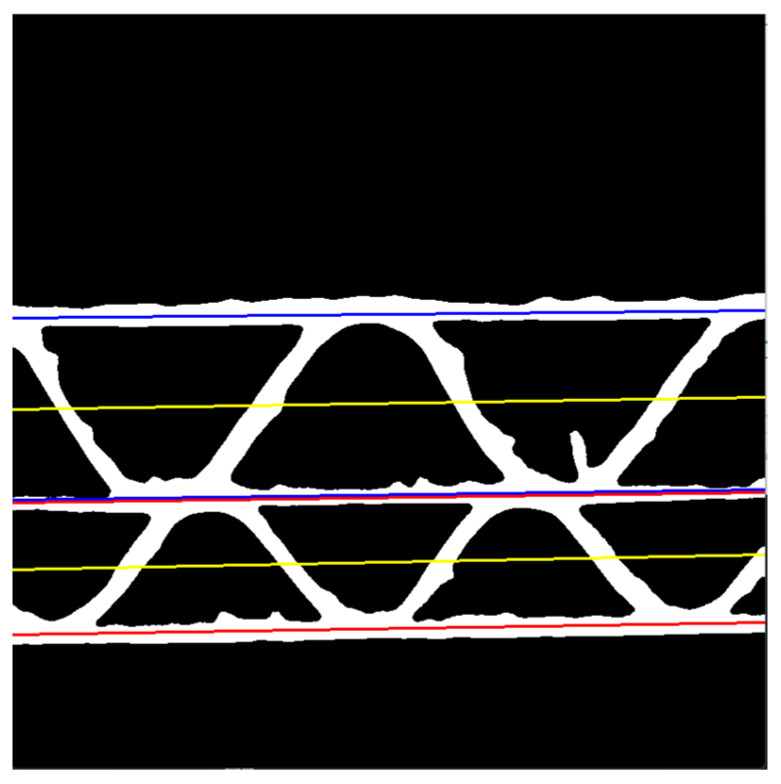
The binary image with boundary lines for limiting the searching areas for ${fluting}_{1}$ (blue lines) and ${fluting}_{2}$ (red lines), and the central lines (yellow lines).

The boundary lines are depicted in Figure 8 in red and blue, while the center lines are presented in yellow. The center lines are approximated as central lines between two boundary lines for each flute, respectively, and can be expressed as

(9) $$y_{center\;1}=a_{center\;1}x+b_{center\;1},$$

(10) $$y_{center\;2}=a_{center\;2}x+b_{center\;2},$$

where $a_{1center}$, $a_{2center},b_{1center},b_{2center}$ are the parameters of the center lines.

The heights of the flutes can be approximated using the following formulas:

(11) $$H_{1}=\frac{1}{NC}\sum_{x=0}^{NC-1}\left|y_{1UBL}\left(x\right)-y_{1LBL}\left(x\right)\right|=\frac{1}{NC}\sum_{x=0}^{NC-1}\left|\left(a_{U}-a_{m}\right)x+b_{U}+b_{US}-b_{m}+b_{mS}\right|,$$

(12) $$H_{2}=\frac{1}{NC}\sum_{x=0}^{NC-1}\left|y_{2UBL}\left(x\right)-y_{2LBL}\left(x\right)\right|=\frac{1}{NC}\sum_{x=0}^{NC-1}\left|\left(a_{m}-a_{L}\right)x+b_{m}+b_{mS}-b_{L}+b_{LS}\right|.$$

It is worth noting that the flute height is equal to two times the amplitude of the sinusoidal function.

#### 2.2.4. Flute Period Searching Range

**Figure 9:**
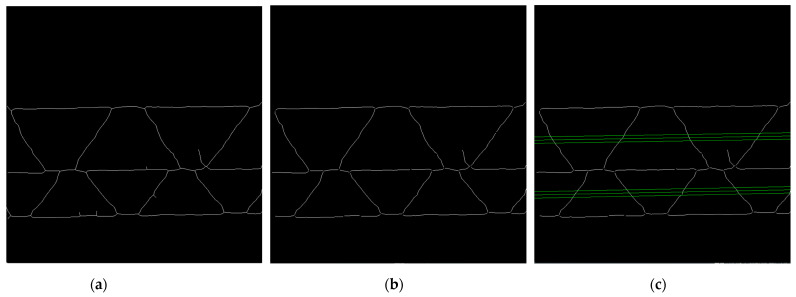
(**a**) Results of the skeletonization process; (**b**) results after the skeletonization process and removing the side branches; (**c**) three parallel green lines for each fluting period limitation.

Next, the binary image is skeletonized. The result of this operation is presented in Figure 9a. The presence of the protruding fibers in the cross-section of the sample can cause some disturbances in the form of side branches in the skeleton. A custom filtering function is applied to the skeletonized image to filter out the unwanted offshoots. As a result, all side branches with contour lengths less than 50 pixels are removed (see Figure 9b).

The limitation of period searching is necessary for the genetic algorithm to provide credible solutions. Values $T_{i\;min}$ and $T_{i\;max}$ (the range of the period searching in pixels for i-th fluting) are based on calculating the distances between the intersection points of the skeleton contours, with three lines parallel to the flutings center line drawn through the flute area (see Figure 9c). The average distance between the successive intersection points and the maximum distance is determined for each fluting. The values $T_{i\;min}=0.5{di}_{i\;av}$ and $T_{max}=3{di}_{i\;max}$ are taken, where ${di}_{i\;av}$ is the average distance between the intersection points for the three parallel lines, and ${di}_{i\;max}$ denotes the maximal value of the distances between these intersection points for i-th fluting. In case of too many disturbances, e.g., due to the presence of long side branches or the critical deformation of the sample, the default values $T_{i\;min}=50$, $T_{i\;max}=800$ are set.

#### 2.2.5. Application of the Genetic Algorithm for the Approximation of Flute Parameters 

The genetic algorithm implemented in [28] enabled the authors to determine sinusoidal function parameters, such as the period and phase shift, to assess the fluting layer geometrical features. In the case of double-wall corrugated board samples, the genetic algorithm ought to be used for each flute independently.

In the searching process for the parameters of the flutes, the following formulas for its approximation were taken into account:

(13) $$y_{{fluting\;}_{i}}=a_{center\;i}x+b_{center\;i}-A_{i}\sin\left(\varphi_{i}+\frac{2\pi }{T_{i}}x\right),$$

where the parameters $a_{center\;i}$, $b_{center\;i}$, and the amplitude $A_{i}=H_{i}/2$ are the fluting parameters determined in the previous stages of the proposed algorithm. The solution from the genetic algorithm is a set of the two following values: phase shift $\varphi_{i}$ and the period $T_{i}$, where *i* denotes the fluting index (i=1,2). Two separate genetic algorithm runs provided an independent set of parameters for each fluting.

**Figure 10:**
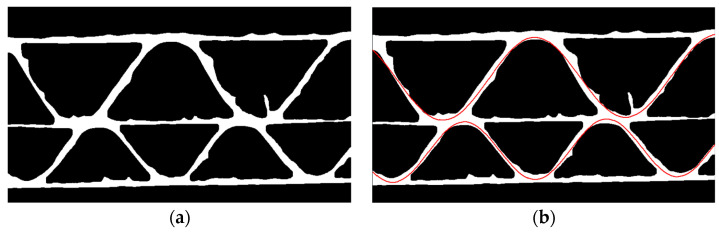
(**a**) The eroded image; (**b**) an example of the results obtained after the optimization processes using the genetic algorithm (red lines).

In each run, the genetic algorithm takes the eroded version of the binary image as an input, as shown in Figure 10a. The main reason for utilizing erosion is to narrow down the flute region, ensuring that the sine function approximation is more precise. The phase shift $\varphi_{i}$ and period $T_{i}$ search are limited by $\varphi_{min}=0$, $\varphi_{max}=2\pi$, $T_{i\;min}$, and $T_{i\;max}$, where *i* denotes the fluting index. The objective function is defined as a total sum of the common pixels for the eroded image and function expressed in Equation (7) or (8) (depending on the analyzed flute) for the given $\varphi_{i}$ and $T_{i}$. 

Applying the genetic algorithm, the following parameters were utilized:

1. Maximal number of iterations: 500;
2. Population size: 100;
3. Mutation probability: 0.15;
4. Elite group ratio (portion of population, which contains the individuals achieved the best performance in the current generation, and are directly copied to the next generation without mutation and crossover): 0.01;
5. Crossover probability: 0.2;
6. Parents portion: 0.2;
7. Crossover type: uniform.

An example of the genetic algorithm result is presented in Figure 10b.

#### 2.2.6. Estimation of the Thickness of the Corrugated Cardboard Layers 

**Figure 11:**
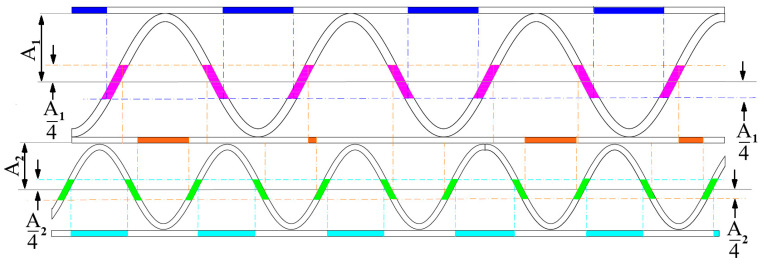
Graphic representation of the liner and flute thickness measurement approach—blue, orange, and turquoise areas—regions for the determination of the upper liner, middle, and lower liners thicknesses. Regions for flute thickness measurements are marked in pink and green.

**Figure 12:**
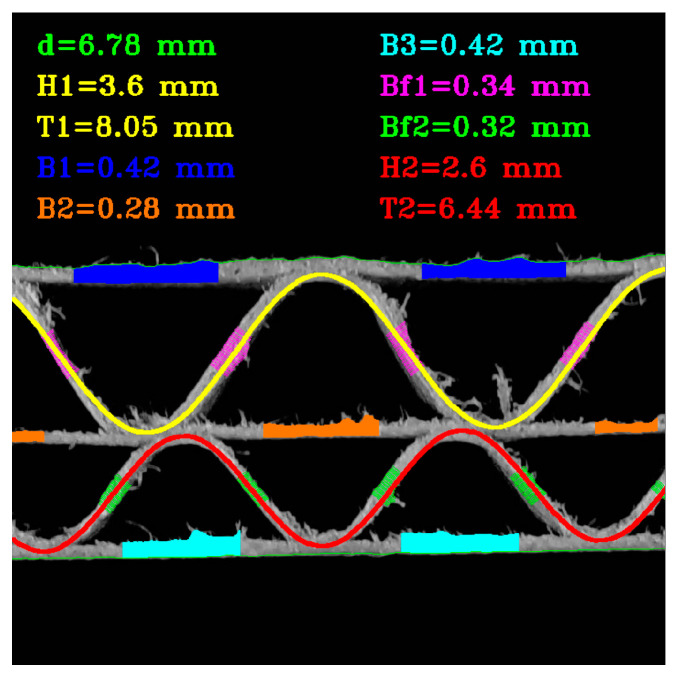
An example of the results obtained by applying the proposed algorithm.

Figure 11 depicts a graphic representation of the idea for measuring the thickness of the paper layers. The approximate location of the flutes in the image is determined based on the sinusoidal function approximations performed in the previous stages of the algorithm. It provides enough information to choose the regions for layer thickness measurements. Areas around layer bonding points are more distorted and usually have a higher number of disturbances, such as protruding fibers. The area of the upper liner, where the thickness can be measured, is marked in blue. A similar region for the middle and bottom liner is marked in orange and turquoise, respectively. The pink and green colors indicate the areas in which the thicknesses of the flutings are determined. Figure 12 shows an example of the result.

## 3. Results

The proposed method allowed the authors to identify the geometrical parameters of any double-wall corrugated board sample in a fully automatic manner. Unfortunately, damaging the structure of the layers or severe cross-section crushing may cause unreliable and false results. 

**Figure 13:**
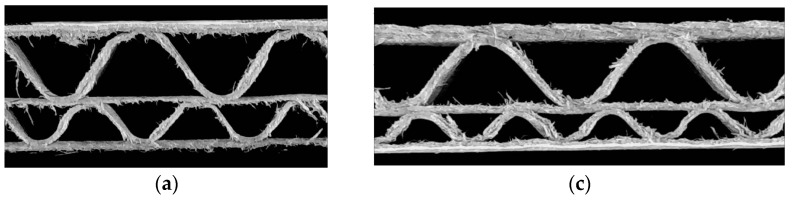

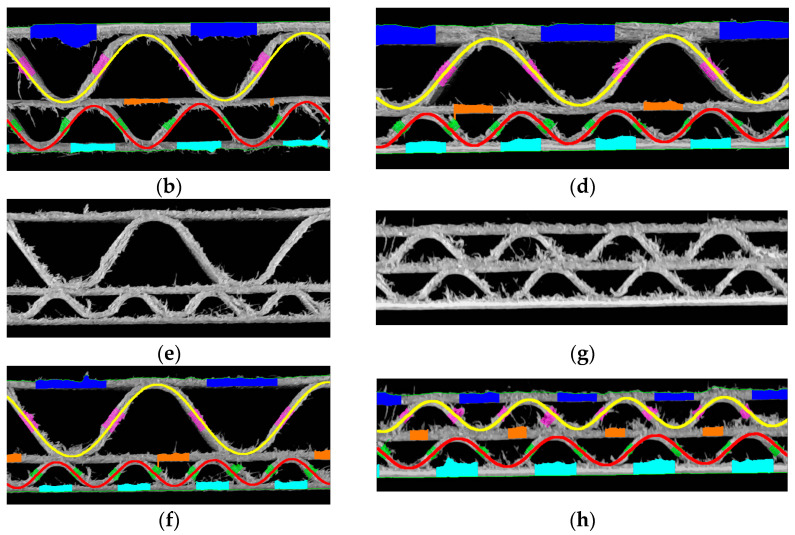
Visualization of the recognized features of the corrugated board: (**a**) flute BC sample; (**b**) the results obtained for the flute BC sample; (**c**) flute EB sample; (**d**) the results obtained for the flute EB sample; (**e**) flute EC sample; (**f**) the results obtained for the flute EC sample; (**g**) flute EE sample; (**h**) the results obtained for the flute EE sample.

Figure 13 presents the results for exemplary corrugated boards samples with BC, EB, EC, and EE flutes. The results in the form of identified geometrical features in pixels and millimeters are summarized in Table 1.

The developed algorithm has limitations similar to those for the single-wall samples presented in [28]. This has to do with the fact that the quality of the sample is the most important factor in terms of the credibility of the obtained results. Every imperfection in the form of, e.g., protruding fibers or corrugated layer deformation resulting from crushing can affect the precision of the method. All samples were cut using an oscillating knife-cutting machine, which creates irregular cut edges and visible shreds of cellulose fibers in all specimens.

**Figure 14:**
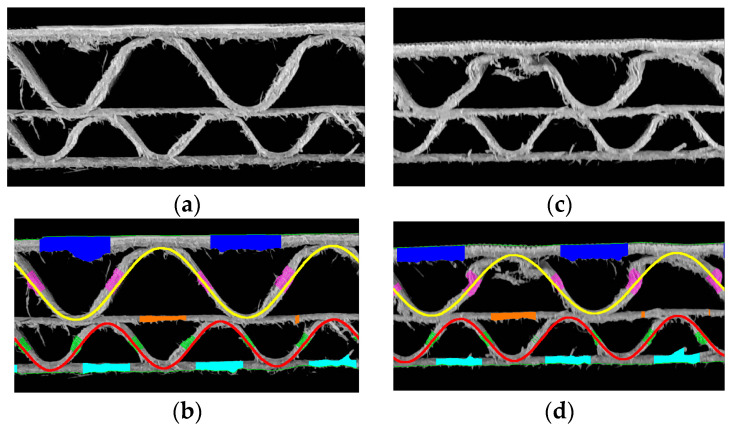
Example of samples with BC flute: (**a**) a reference sample; (**b**) the results of identification for the reference sample; (**c**) a crushed sample with many jagged edges; (**d**) the results of identification for the crushed sample.

In Figure 14, two BC flute samples are presented. The one on the left is not damaged in any way by creasing. The sample on the right is crushed stochastically with a visible change in fluting shape. It can be noticed that the algorithm still solves the sinusoidal function approximation, but does not reflect the deformed corrugated layer shape. In this case, the flute period and height are not correctly measured, but can be used to estimate the actual flute type of the crushed sample. Table 2 contains the identified parameters for these two samples.

**Figure 15:**
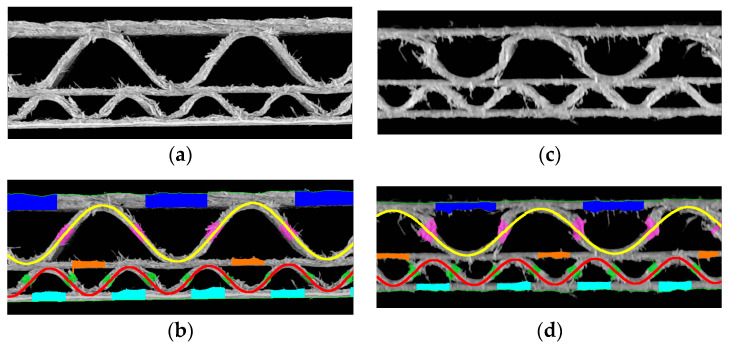
EB flute example: (**a**) a cardboard without damage; (**b**) the results of geometrical features identification for the sample without damage; (**c**) a damaged sample; (**d**) the results of geometrical features identification for the damaged sample.

Figure 15 shows two samples of the corrugated board with the EB flute and the results of their geometrical features identification. The corrugated board on the left is without any damage and the one on the right demonstrates damage in the form of a crushed flute and multiple protruding cellulose fibers. The identified geometrical parameters for both samples with EB flutes are presented in Table 3.

**Figure 16:**
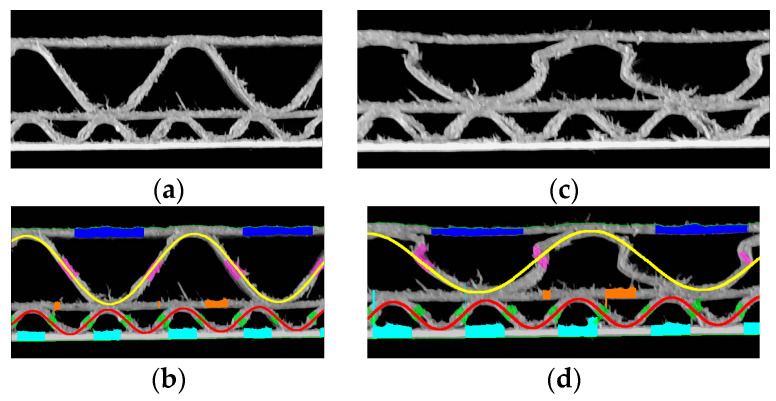
Example of samples with the EC flute: (**a**) a reference sample; (**b**) the results of identification for the reference sample; (**c**) a crushed sample with many jagged edges; (**d**) the results of identification for the crushed sample.

The examples presented in Figure 16 depict two EC flute specimens cut out from the same cardboard sheet. The one on the left is a reference sample, whereas on the right, the sample with the cutting edge is shown, which was crushed by creasing. The results of processing both images are visible in Table 4. Severe deformation of the higher fluting is again causing a low-precision sine approximation. Period and height measurements in the case of lower flutes are more reliable since the creasing is less visible in the cross-section. On the other hand, in the case of E flutes, the presence of noise in the form of jagged edges and cellulose fibers intensifies due to the reduced distance between each layer. Hence, difficulties with obtaining reliable layer thickness measurements can occur. 

**Figure 17:**
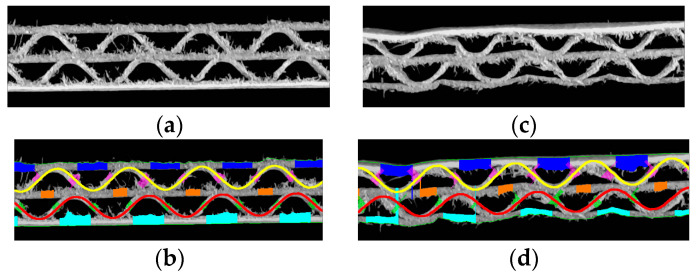
EE flute example: (**a**) a reference sample; (**b**) the results of identification for the reference sample; (**c**) a damaged sample; (**d**) the results of identification for the damaged sample.

The last example is shown in Figure 17, where two samples of corrugated boards with EE flutes are presented. Since the sample combines two of the lowest possible flutes presented, it is supposedly the most difficult to analyze. The crease in such cardboard is less visible (example on the right). Nevertheless, as the geometrical dimensions of the cross-section are reduced, the most significant trouble is measuring the thickness of individual paper layers. As can be noticed in the exemplary results in Figure 17b, the measured liners and flute areas are not marked correctly; hence, the measurements are flawed. This complex problem is further elaborated on in the discussion section. Table 5 presents the results of the parameters identification for both samples of corrugated boards with EE flutes.

The presented results are only a small part of the sample images processed during this research. A total number of 310 different images were processed with the proposed algorithm. The database consisted of reference, stochastically deformed (different forces applied at different locations), and crushed via creasing corrugated board samples.

## 4. Discussion

In the results section, some exemplary outcomes from the algorithm for various cardboard samples were presented. In order to evaluate this, in some way, innovative method for identifying cross-section corrugated board geometrical features, the following section takes up the critical discussion of the results and the limitations of the proposed approach.

As previously emphasized, the method’s effectiveness relies significantly on the precision of sample cutting. The cellulose shreds and protruding fibers visible in each cross-section are a side effect of using an oscillating knife as a cutting tool. Since their presence affects the outcome to a great extent, excising the samples by using, e.g., a laser cutter, is expected to provide more trustworthy results. Another important factor that affected the accuracy of the algorithm were the damages introduced into the corrugated board in the process of stochastic deformation and creasing.

As described in [28], the delamination of layers, corrugated layer crushing, and the presence of jagged edges create noticeable difficulties in determining the thickness of each paper layer. These difficulties can also be found in the presented examples, e.g., in Figure 17d (visibly flawed liners thickness measurement caused by cellulose fibers) and Figure 16d (flawed flutes thickness measurement caused by its high level of crush). The most reliable geometric parameters obtained from the algorithm seem always to be the period of the corrugated layer. Even in cases of an inaccurate fit of the approximating function, which was assumed as a sine function, to the shape of the visibly crushed fluting, the period measurement can be considered a reasonable approximation (Figure 14d and Figure 16d). In these scenarios, the heights of the corrugated layers are not entirely credible and can be considered as auxiliary indicators.

**Figure 18:**
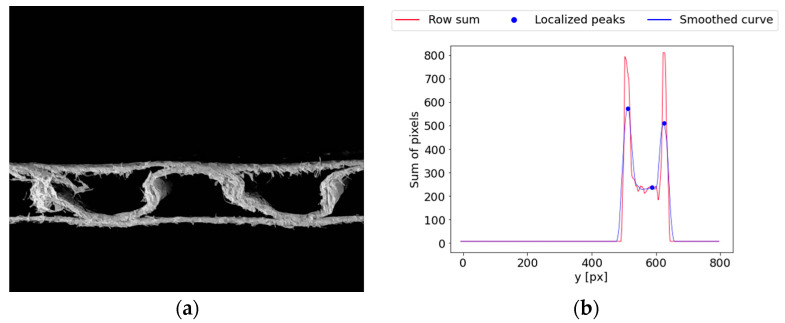
Example of the error in recognizing the number of layers in the corrugated board sample: (**a**) a single-wall C-flute sample with a visible hank of cellulose fibers; (**b**) a smoothed row–sum curve of the image with wrongly localized peaks, potentially reflecting the liners of the corrugated board.

Another problem can occur during the analysis of the cross-section of different samples. Figure 18a shows a single-wall C-flute sample with a visible hank of cellulose fibers on the left side of the image in the region between liners. Figure 18b depicts a smoothed row sum curve with detected peaks for this sample. Three local maximums were recognized in the first stages of the algorithm. Usually, this would indicate a double-wall cardboard type, which, in this case, is obviously incorrect. The workflow of the algorithm differs significantly for single- and double-wall corrugated board samples. Therefore, the obtained results would be unfit for further use.

**Figure 19:**
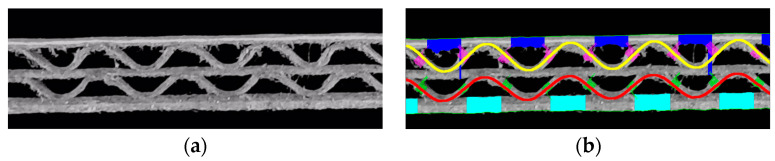
Error in the measurements of the middle liner thickness due to in-phase composition of the corrugated layers: (**a**) flute-EE sample; (**b**) incomplete results obtained for the flute-EE sample.

Difficulty in the measurements of the liner thickness can also be caused by factors other than cross-section noise. Figure 19 depicts a relatively common (among samples processed in the research) case of both flutes being in phase with each other, meaning the phase shifts for both corrugated layers are almost identical. Considering that the regions for measuring each layer thickness are calculated based on the approach presented in Figure 11, it is impossible for the algorithm to obtain a credible area for middle liner thickness measurements. This results in the method providing an incomplete set of geometrical features of the sample.

**Figure 20:**
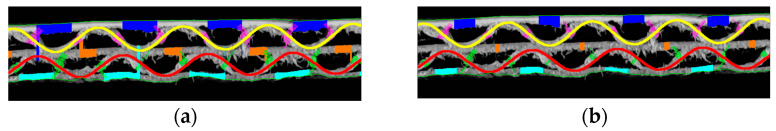
(**a**) The original example (very weak effect) and (**b**) the results of the same image after limiting the layer thickness measurement areas.

The biggest issue with the proposed method is commonly visible in processing microwave samples. The layer thickness measurement was flawed in most of the processed flute-EE corrugated board images. This is due to interference (jagged fibers) concerning the size of the area between the layers. A possible solution to this problem may be to limit the regions for measuring thicknesses for each layer. The different conditions for microwave flutes adopted in the approach presented in Figure 11 can improve the efficiency of the algorithm. Figure 20a,b present results for the same image before and after limiting the areas, respectively. The improved approach provides significantly better results. Nonetheless, some errors, especially in flute thickness measurements, still occur.

## 5. Conclusions

In this paper, the method for identifying the geometric features of double-wall corrugated board cross-sections is presented. The cross-section images were collected using a device first introduced in [28], where a similar algorithm was successfully applied for single-wall corrugated cardboard. This enhanced method primarily yields reliable results for five-ply samples, and is widely used in the packaging industry. The geometrical characteristics of the sample were determined through complex image processing techniques and genetic algorithms. The use of local algorithms, such as gradient-based, is not feasible for the specific nature of the optimization problem.

The method’s effectiveness and reliability largely depend on the quality of the sample. The fit of the approximation function to the flute’s shape, in terms of height and period, is strongly affected by the extent of the cardboard’s cross-section crushing. Additionally, cutting corrugated boards on an oscillating knife-cutting machine, a common industry practice, aligns well with this method, enabling its practical applications.

In summary, while the method has some limitations, improvements seem feasible in the near future. A quick and relatively simple algorithm for extracting geometrical features could be a crucial step in automating the modeling of corrugated board structures. In future studies, other evolutionary algorithms can also be utilized to find the fluting shape of corrugated cardboard, and the results presented in this work can serve as a reference.

## Figures and Tables

**Table 1:** Identification results of the geometrical features for the corrugated boards with flutes BC, EB, EC, and EE.

	Flute BC		Flute EB		Flute EC		Flute EE	
	[px]	[mm]	[px]	[mm]	[px]	[mm]	[px]	[mm]
${flute}_{1}$ height	165	3.13	133	2.53	175	3.34	59	1.12
${flute}_{1}$ period	396	7.48	347	6.56	423	7.99	190	3.59
${flute}_{2}$ height	107	2.03	64	1.22	64	1.22	58	1.1
${flute}_{2}$ period	265	5.01	184	3.48	194	3.67	192	3.63
Board thickness	312	5.93	245	4.66	276	5.24	161	3.06
Upper liner thickness	39	0.74	33	0.63	20	0.38	18	0.34
Middle liner thickness	12	0.23	15	0.28	19	0.36	18	0.34
Bottom liner thickness	17	0.32	21	0.4	17	0.32	25	0.47
${flute}_{1}$ thickness	22	0.42	19	0.36	20	0.38	16	0.30
${flute}_{2}$ thickness	17	0.32	17	0.32	20	0.38	13	0.25

**Table 2:** Identification results of the geometrical features for the corrugated board with flute BC (reference and crushed samples).

	Flute BC (Reference)		Flute BC (Crushed)	
	[px]	[mm]	[px]	[mm]
${flute}_{1}$ height	165	3.13	144	2.74
${flute}_{1}$ period	396	7.48	393	7.43
${flute}_{2}$ height	107	2.03	104	1.98
${flute}_{2}$ period	265	5.01	262	4.95
Board thickness	312	5.93	286	5.43
Upper liner thickness	39	0.74	35	0.67
Middle liner thickness	12	0.23	15	0.28
Bottom liner thickness	17	0.32	18	0.34
${flute}_{1}$ thickness	22	0.42	23	0.44
${flute}_{2}$ thickness	17	0.32	15	0.28

**Table 3:** Identification results of the geometrical features for the corrugated board with the flute EB (reference and crushed samples).

	Flute EB (Reference)		Flute EB (Crushed)	
	[px]	[mm]	[px]	[mm]
${flute}_{1}$ height	133	2.53	107	2.03
${flute}_{1}$ period	347	6.56	343	6.48
${flute}_{2}$ height	64	1.22	60	1.14
${flute}_{2}$ period	184	3.48	190	3.59
Board thickness	245	4.66	206	3.91
Upper liner thickness	33	0.63	20	0.38
Middle liner thickness	15	0.28	12	0.23
Bottom liner thickness	21	0.4	17	0.32
${flute}_{1}$ thickness	19	0.36	24	0.46
${flute}_{2}$ thickness	17	0.32	19	0.36

**Table 4:** Identification results of the geometrical features for the corrugated board with flute EC (reference and crushed samples).

	Flute EC (Reference)		Flute EC (Crushed)	
	[px]	[mm]	[px]	[mm]
${flute}_{1}$ height	177	3.36	124	2.36
${flute}_{1}$ period	430	8.13	456	8.62
${flute}_{2}$ height	62	1.18	59	1.12
${flute}_{2}$ period	193	3.65	189	3.57
Board thickness	284	5.4	226	4.29
Upper liner thickness	22	0.42	16	0.30
Middle liner thickness	19	0.36	20	0.38
Bottom liner thickness	25	0.47	26	0.49
${flute}_{1}$ thickness	20	0.38	20	0.38
${flute}_{2}$ thickness	19	0.36	20	0.38

**Table 5:** Identification results of the geometrical features for the corrugated board with EE flutes (reference and crushed samples).

	Flute EE (Reference)		Flute EE (Crushed)	
	[px]	[mm]	[px]	[mm]
${flute}_{1}$ height	59	1.12	62	1.18
${flute}_{1}$ period	192	3.63	189	3.57
${flute}_{2}$ height	58	1.1	52	0.99
${flute}_{2}$ period	191	3.61	186	3.52
Board thickness	161	3.06	146	2.77
Upper liner thickness	18	0.34	29	0.55
Middle liner thickness	18	0.34	20	0.38
Bottom liner thickness	25	0.47	15	0.28
${flute}_{1}$ thickness	16	0.30	19	0.36
${flute}_{2}$ thickness	13	0.25	16	0.30

## Data Availability

Data are available on request.
